# 2,4,6-Tri-*p*-tolyl­pyridine

**DOI:** 10.1107/S160053680901931X

**Published:** 2009-05-29

**Authors:** Si-Ping Tang, Dai-Zhi Kuang, Yong-Lan Feng, Man-Sheng Chen, Wei Li

**Affiliations:** a Key Laboratory of Functional Organometallic Materials, Hengyang Normal University, Hengyang, Hunan 421008, People’s Republic of China

## Abstract

In the title compound, C_26_H_23_N, the complete molecule is generated by crystallographic mirror symmetry, with the N atom and four C atoms lying on the reflection plane. The dihedral angles between the pyridine ring and pendant benzene rings are 2.9 (1), 14.1 (1) and 14.1 (1)°. Neighbouring mol­ecules are stabilized through inter­molecular π–π inter­actions along the *c* axis [centroid-to-centroid distance = 3.804 (2) Å], forming one-dimensional chains.

## Related literature

For the syntheses of related 2,4,6-triaryl­pyridine compounds, see: Hou *et al.* (2005 ▶); Huang *et al.* (2005 ▶); Tewari *et al.* (1981 ▶); Yang *et al.* (2005 ▶).
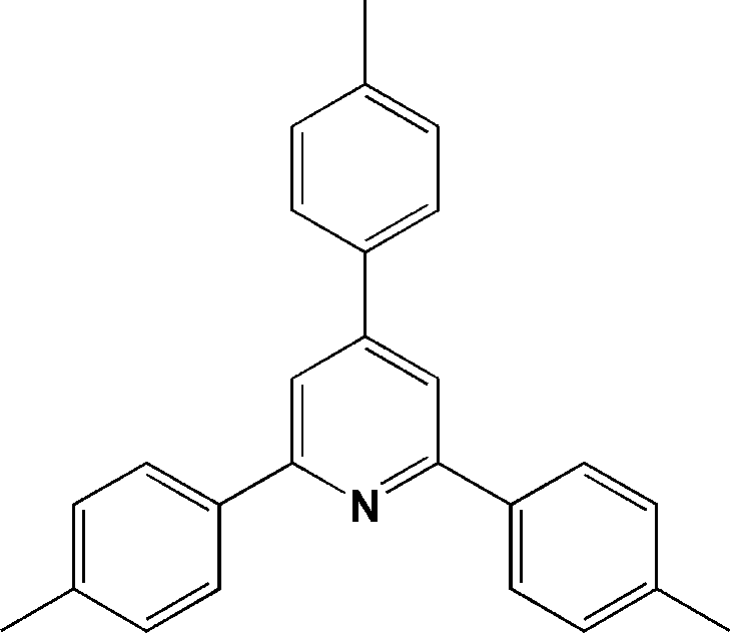


## Experimental

### 

#### Crystal data

C_26_H_23_N
*M* *_r_* = 349.45
Orthorhombic, 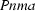

*a* = 15.337 (5) Å
*b* = 20.778 (7) Å
*c* = 6.322 (2) Å
*V* = 2014.8 (11) Å^3^
*Z* = 4
Mo *K*α radiation
μ = 0.07 mm^−1^
*T* = 295 K
0.24 × 0.16 × 0.15 mm

#### Data collection

Bruker SMART APEX area-detector diffractometer
Absorption correction: multi-scan (*SADABS*; Sheldrick, 1996 ▶) *T*_min_ = 0.975, *T*_max_ = 0.986
7912 measured reflections
2037 independent reflections
924 reflections with *I* > 2σ(*I*)
*R*_int_ = 0.067

#### Refinement

*R*[*F* ^2^ > 2σ(*F* ^2^)] = 0.139
*wR*(*F* ^2^) = 0.342
*S* = 1.26
2037 reflections
132 parameters
47 restraints
H-atom parameters constrained
Δρ_max_ = 0.27 e Å^−3^
Δρ_min_ = −0.20 e Å^−3^

### 

Data collection: *SMART* (Bruker, 2002 ▶); cell refinement: *SAINT* (Bruker, 2002 ▶); data reduction: *SAINT*; program(s) used to solve structure: *SHELXS97* (Sheldrick, 2008 ▶); program(s) used to refine structure: *SHELXL97* (Sheldrick, 2008 ▶); molecular graphics: *SHELXTL* (Sheldrick, 2008 ▶); software used to prepare material for publication: *SHELXTL*.

## Supplementary Material

Crystal structure: contains datablocks I, global. DOI: 10.1107/S160053680901931X/at2790sup1.cif

Structure factors: contains datablocks I. DOI: 10.1107/S160053680901931X/at2790Isup2.hkl

Additional supplementary materials:  crystallographic information; 3D view; checkCIF report

## Figures and Tables

**Fig. 1.**
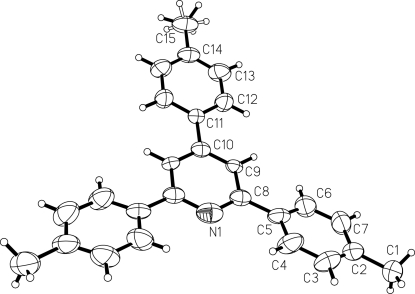
The title molecule with displacement ellipsoids drawn at the 30% probability level, and H atoms as spheres of arbitrary radius.

## supplementary crystallographic information

### Comment

2,4,6-Triarylpyridines are used as good building blocks in supramolecular chemistry because of their stacking ability, directional H-bonding and coordination, and which have also been prepared by many procedures (Hou *et al.*, 2005; Huang *et al.*, 2005; Tewari *et al.*, 1981; Yang *et al.*, 2005). We here reported the synthesis and crystal structure of 2,4,6-tri-*p*-tolylpyridine.

As shown in Fig.1, the title compound is a neutral organic molecule with a mirror symmetry through the methyl C15 atom and N1 atom of the central pyridine. The central pyridine is almost coplanar with the C_11-14_ benzene ring with a dihedral angle of 2.9 (1) °, however, which form bigger dihedral angles of 14.1 (1) ° with the other two outer benzene rings, thus the whole molecule is nonplanar. In the crystal packing, neighboring molecules form intermolecular π–π interactions with the centroid- to-centroid distances of 3.804 (2) Å to give a one-dimensional chain along the *c*-axis.

### Experimental

The title compound was synthesized with a modified procedure (Yang *et al.*, 2005). A mixture of 5-tri-*p*-tolyl-pentane-1,5-dione (1.85 g, 5 mmol), ammonium acetate (3.85 g, 50 mmol) and ethanol (60 mL) was refluxed for 20 h. Upon cooling to room temperature, a precipitate was filtered, washed with ethanol/water (1:1) and dried to afford the product, purified by column chromatography on silica with petroleum/ethyl acetate. A white solid was obtained and was further recrystallized from ethanol to give colourless crystals [yield: 0.85 g, 48.6%].

### Refinement

The carbon-bound H atoms were placed at calculated positions (C—H = 0.93 and 0.96 Å) and refined as riding, with *U*(H) = 1.2*U*_eq_(C) for benzenel H atoms, and C—H = 0.96 Å and *U*_iso_ = 1.5*U*_eq_ (C) for methyl H atoms.

### Crystal data

**Table:**

C_26_H_23_N	*F*(000) = 744
*M**_r_* = 349.45	*D*_x_ = 1.152 Mg m^−^^3^
Orthorhombic, *P**n**m**a*	Mo *K*α radiation, λ = 0.71073 Å
Hall symbol: -P 2ac 2n	Cell parameters from 562 reflections
*a* = 15.337 (5) Å	θ = 2.7–22.4°
*b* = 20.778 (7) Å	µ = 0.07 mm^−^^1^
*c* = 6.322 (2) Å	*T* = 295 K
*V* = 2014.8 (11) Å^3^	Prism, colourless
*Z* = 4	0.24 × 0.16 × 0.15 mm

### Data collection

**Table:**

Bruker SMART APEX area-detector diffractometer	2037 independent reflections
Radiation source: fine-focus sealed tube	924 reflections with *I* > 2σ(*I*)
graphite	*R*_int_ = 0.067
φ and ω scans	θ_max_ = 26.0°, θ_min_ = 2.0°
Absorption correction: multi-scan (*SADABS*; Sheldrick, 1996)	*h* = −17→18
*T*_min_ = 0.975, *T*_max_ = 0.986	*k* = −20→25
7912 measured reflections	*l* = −7→5

### Refinement

**Table:**

Refinement on *F*^2^	Primary atom site location: structure-invariant direct methods
Least-squares matrix: full	Secondary atom site location: difference Fourier map
*R*[*F*^2^ > 2σ(*F*^2^)] = 0.139	Hydrogen site location: inferred from neighbouring sites
*wR*(*F*^2^) = 0.342	H-atom parameters constrained
*S* = 1.26	*w* = 1/[σ^2^(*F*_o_^2^) + (0.0923*P*)^2^ + 1.1054*P*] where *P* = (*F*_o_^2^ + 2*F*_c_^2^)/3
2037 reflections	(Δ/σ)_max_ < 0.001
132 parameters	Δρ_max_ = 0.27 e Å^−^^3^
47 restraints	Δρ_min_ = −0.20 e Å^−^^3^

### Special details

**Table:**

Geometry. All esds (except the esd in the dihedral angle between two l.s. planes) are estimated using the full covariance matrix. The cell esds are taken into account individually in the estimation of esds in distances, angles and torsion angles; correlations between esds in cell parameters are only used when they are defined by crystal symmetry. An approximate (isotropic) treatment of cell esds is used for estimating esds involving l.s. planes.
Refinement. Refinement of F^2^ against ALL reflections. The weighted R-factor wR and goodness of fit S are based on F^2^, conventional R-factors R are based on F, with F set to zero for negative F^2^. The threshold expression of F^2^ > 2sigma(F^2^) is used only for calculating R-factors(gt) etc. and is not relevant to the choice of reflections for refinement. R-factors based on F^2^ are statistically about twice as large as those based on F, and R- factors based on ALL data will be even larger.

### Fractional atomic coordinates and isotropic or equivalent isotropic displacement parameters (Å^2^)

**Table:**

	*x*	*y*	*z*	*U*_iso_*/*U*_eq_	Occ. (<1)
C1	0.4178 (6)	0.5536 (4)	0.7562 (16)	0.180 (4)	
H1A	0.4197	0.5501	0.9076	0.270*	
H1B	0.3776	0.5869	0.7165	0.270*	
H1C	0.4749	0.5639	0.7038	0.270*	
C2	0.3882 (5)	0.4900 (4)	0.6625 (15)	0.139 (3)	
C3	0.3948 (6)	0.4331 (5)	0.7686 (15)	0.158 (3)	
H3	0.4171	0.4334	0.9053	0.190*	
C4	0.3696 (5)	0.3750 (4)	0.6812 (13)	0.143 (3)	
H4	0.3750	0.3378	0.7624	0.172*	
C5	0.3376 (4)	0.3699 (4)	0.4833 (11)	0.098 (2)	
C6	0.3283 (6)	0.4267 (5)	0.3786 (13)	0.139 (3)	
H6	0.3050	0.4261	0.2428	0.167*	
C7	0.3522 (6)	0.4853 (4)	0.4650 (15)	0.163 (4)	
H7	0.3435	0.5226	0.3866	0.196*	
C8	0.3116 (4)	0.3075 (3)	0.3878 (9)	0.0859 (18)	
C9	0.2622 (3)	0.3058 (2)	0.2112 (8)	0.0646 (14)	
H9	0.2455	0.3443	0.1479	0.077*	
N1	0.3373 (5)	0.2500	0.4758 (13)	0.123 (3)	
C10	0.2366 (5)	0.2500	0.1249 (12)	0.075 (2)	
C11	0.1802 (4)	0.2500	−0.0662 (12)	0.0673 (19)	
C12	0.1525 (4)	0.3049 (3)	−0.1594 (11)	0.107 (2)	
H12	0.1713	0.3442	−0.1057	0.128*	
C13	0.0972 (5)	0.3044 (3)	−0.3317 (11)	0.121 (2)	
H13	0.0794	0.3438	−0.3873	0.145*	
C14	0.0676 (6)	0.2500	−0.4240 (15)	0.103 (3)	
C15	0.0081 (6)	0.2500	−0.6115 (15)	0.133 (3)	
H15A	0.0335	0.2753	−0.7227	0.199*	0.50
H15B	−0.0472	0.2681	−0.5722	0.199*	0.50
H15C	−0.0001	0.2066	−0.6600	0.199*	0.50

### Atomic displacement parameters (Å^2^)

**Table:**

	*U*^11^	*U*^22^	*U*^33^	*U*^12^	*U*^13^	*U*^23^
C1	0.158 (7)	0.165 (7)	0.217 (9)	0.049 (6)	−0.066 (7)	−0.093 (7)
C2	0.111 (5)	0.149 (7)	0.157 (7)	0.030 (5)	−0.054 (5)	−0.053 (5)
C3	0.153 (5)	0.181 (7)	0.141 (6)	−0.015 (5)	−0.063 (5)	−0.029 (5)
C4	0.142 (5)	0.165 (6)	0.122 (5)	−0.027 (4)	−0.049 (5)	−0.006 (5)
C5	0.074 (4)	0.142 (5)	0.078 (4)	0.003 (4)	−0.022 (3)	−0.008 (4)
C6	0.157 (7)	0.141 (7)	0.119 (7)	0.036 (6)	−0.052 (5)	−0.024 (6)
C7	0.186 (9)	0.132 (7)	0.171 (9)	0.057 (6)	−0.061 (8)	−0.042 (6)
C8	0.063 (3)	0.120 (5)	0.075 (4)	0.002 (4)	0.002 (3)	0.001 (4)
C9	0.054 (3)	0.080 (3)	0.060 (3)	0.011 (3)	−0.013 (3)	−0.001 (3)
N1	0.089 (6)	0.180 (9)	0.098 (6)	0.000	0.011 (5)	0.000
C10	0.055 (4)	0.103 (6)	0.065 (5)	0.000	0.011 (4)	0.000
C11	0.060 (4)	0.076 (5)	0.066 (5)	0.000	−0.002 (4)	0.000
C12	0.119 (5)	0.092 (4)	0.110 (5)	−0.002 (4)	−0.037 (4)	0.003 (4)
C13	0.114 (5)	0.141 (6)	0.108 (5)	0.004 (4)	−0.038 (4)	0.031 (4)
C14	0.078 (5)	0.152 (7)	0.079 (5)	0.000	−0.020 (4)	0.000
C15	0.098 (6)	0.216 (9)	0.085 (6)	0.000	−0.022 (5)	0.000

### Geometric parameters (Å, °)

**Table:**

C1—C2	1.518 (10)	C9—C10	1.340 (6)
C1—H1A	0.9600	C9—H9	0.9300
C1—H1B	0.9600	N1—C8^i^	1.375 (5)
C1—H1C	0.9600	C10—C9^i^	1.340 (6)
C2—C3	1.361 (8)	C10—C11	1.486 (10)
C2—C7	1.369 (8)	C11—C12^i^	1.352 (6)
C3—C4	1.384 (10)	C11—C12	1.352 (6)
C3—H3	0.9300	C12—C13	1.381 (8)
C4—C5	1.348 (9)	C12—H12	0.9300
C4—H4	0.9300	C13—C14	1.352 (6)
C5—C6	1.361 (9)	C13—H13	0.9300
C5—C8	1.484 (8)	C14—C13^i^	1.352 (6)
C6—C7	1.384 (9)	C14—C15	1.495 (12)
C6—H6	0.9300	C15—H15A	0.9600
C7—H7	0.9300	C15—H15B	0.9600
C8—C9	1.350 (7)	C15—H15C	0.9600
C8—N1	1.375 (5)		
			
C2—C1—H1A	109.5	N1—C8—C5	121.1 (6)
C2—C1—H1B	109.5	C10—C9—C8	121.5 (6)
H1A—C1—H1B	109.5	C10—C9—H9	119.2
C2—C1—H1C	109.5	C8—C9—H9	119.2
H1A—C1—H1C	109.5	C8^i^—N1—C8	120.6 (9)
H1B—C1—H1C	109.5	C9—C10—C9^i^	119.8 (7)
C3—C2—C7	114.7 (9)	C9—C10—C11	120.1 (4)
C3—C2—C1	122.7 (8)	C9^i^—C10—C11	120.1 (4)
C7—C2—C1	122.6 (9)	C12^i^—C11—C12	115.0 (8)
C2—C3—C4	122.7 (8)	C12^i^—C11—C10	122.5 (4)
C2—C3—H3	118.7	C12—C11—C10	122.5 (4)
C4—C3—H3	118.7	C11—C12—C13	122.1 (6)
C5—C4—C3	122.7 (9)	C11—C12—H12	118.9
C5—C4—H4	118.6	C13—C12—H12	118.9
C3—C4—H4	118.6	C14—C13—C12	123.5 (7)
C4—C5—C6	114.9 (8)	C14—C13—H13	118.2
C4—C5—C8	123.0 (7)	C12—C13—H13	118.2
C6—C5—C8	122.1 (6)	C13—C14—C13^i^	113.6 (9)
C5—C6—C7	122.9 (8)	C13—C14—C15	123.2 (4)
C5—C6—H6	118.5	C13^i^—C14—C15	123.2 (4)
C7—C6—H6	118.5	C14—C15—H15A	109.5
C2—C7—C6	122.0 (9)	C14—C15—H15B	109.5
C2—C7—H7	119.0	H15A—C15—H15B	109.5
C6—C7—H7	119.0	C14—C15—H15C	109.5
C9—C8—N1	118.2 (7)	H15A—C15—H15C	109.5
C9—C8—C5	120.6 (5)	H15B—C15—H15C	109.5
			
C7—C2—C3—C4	1.9 (14)	C5—C8—C9—C10	−178.7 (6)
C1—C2—C3—C4	−178.5 (8)	C9—C8—N1—C8^i^	−1.3 (11)
C2—C3—C4—C5	0.8 (15)	C5—C8—N1—C8^i^	178.9 (5)
C3—C4—C5—C6	−2.7 (12)	C8—C9—C10—C9^i^	−1.8 (10)
C3—C4—C5—C8	179.1 (7)	C8—C9—C10—C11	178.3 (5)
C4—C5—C6—C7	1.8 (12)	C9—C10—C11—C12^i^	−179.9 (6)
C8—C5—C6—C7	180.0 (7)	C9^i^—C10—C11—C12^i^	0.2 (10)
C3—C2—C7—C6	−2.8 (14)	C9—C10—C11—C12	−0.2 (10)
C1—C2—C7—C6	177.6 (8)	C9^i^—C10—C11—C12	179.9 (6)
C5—C6—C7—C2	1.0 (15)	C12^i^—C11—C12—C13	2.5 (12)
C4—C5—C8—C9	164.8 (6)	C10—C11—C12—C13	−177.3 (6)
C6—C5—C8—C9	−13.2 (10)	C11—C12—C13—C14	−1.3 (12)
C4—C5—C8—N1	−15.4 (10)	C12—C13—C14—C13^i^	0.0 (15)
C6—C5—C8—N1	166.6 (7)	C12—C13—C14—C15	−179.6 (8)
N1—C8—C9—C10	1.5 (9)		

Symmetry codes: (i) *x*, −*y*+1/2, *z*.
